# Autonomous clocks that regulate organelle biogenesis, cytoskeletal organization, and intracellular dynamics

**DOI:** 10.7554/eLife.72104

**Published:** 2021-09-29

**Authors:** Mohammad Mofatteh, Fabio Echegaray-Iturra, Andrew Alamban, Francesco Dalla Ricca, Anand Bakshi, Mustafa G Aydogan

**Affiliations:** 1 Department of Biochemistry and Biophysics, University of California, San Francisco San Francisco United States; Fred Hutchinson Cancer Research Center United States; Fred Hutchinson Cancer Research Center United States

**Keywords:** biological timing, autonomous clocks, cell cycle, circadian clock, organelle dynamics, cytoskeleton

## Abstract

How do cells perceive time? Do cells use temporal information to regulate the production/degradation of their enzymes, membranes, and organelles? Does controlling biological time influence cytoskeletal organization and cellular architecture in ways that confer evolutionary and physiological advantages? Potential answers to these fundamental questions of cell biology have historically revolved around the discussion of ‘master’ temporal programs, such as the principal cyclin-dependent kinase/cyclin cell division oscillator and the circadian clock. In this review, we provide an overview of the recent evidence supporting an emerging concept of ‘autonomous clocks,’ which under normal conditions can be *entrained* by the cell cycle and/or the circadian clock to run at their pace, but can also run independently to serve their functions if/when these major temporal programs are halted/abrupted. We begin the discussion by introducing recent developments in the study of such clocks and their roles at different scales and complexities. We then use current advances to elucidate the logic and molecular architecture of temporal networks that comprise autonomous clocks, providing important clues as to how these clocks may have evolved to run independently and, sometimes at the cost of redundancy, have strongly coupled to run under the full command of the cell cycle and/or the circadian clock. Next, we review a list of important recent findings that have shed new light onto potential hallmarks of autonomous clocks, suggestive of prospective theoretical and experimental approaches to further accelerate their discovery. Finally, we discuss their roles in health and disease, as well as possible therapeutic opportunities that targeting the autonomous clocks may offer.

## The emerging concept of autonomous clocks and their mechanisms at different scales and complexities

What is an ‘autonomous clock’? An introduction to the syntax of this emerging term is essential as various fields of biological timing studies have taken on the words ‘autonomous’ and ‘clock’ in fundamentally different ways. For example, the word ‘clock’ has historically been used to define the time-keeping machinery that regulates the circadian rhythms. In this context, a clock has a strict meaning, in that it refers to an oscillator that is able to (1) free-run, (2) receive entrainment cues from the environment, and (3) compensate for changes in temperature (Bell-Pedersen et al., 2005; Rosbash, 2009; Tauber et al., 2004; Young and Kay, 2001). The emerging concept of ‘autonomous clocks,’ however, adopts the word ‘clock’ as an umbrella term for ‘timing mechanisms’ without discriminating between the nomenclature of timers, hour-glass mechanisms, or different types of oscillators (see Gliech and Holland, 2020 for finer details of these terminologies). Similarly, the word ‘autonomous’ has been used in the circadian field to describe the self-sustained 24 hr rhythms in individual cells (i.e., ‘cell-intrinsic’), as opposed to rhythms that are ‘cell non-autonomous,’ originating from synchronization at the population level via systemic cues (such as changes in temperature or feeding frequency). In contrast, an ‘autonomous system’ in mathematics connotates a set of differential equations that does not explicitly depend on the independent variable – when the variable is time, the system becomes time-invariant. Meanwhile, the ‘autonomy’ (of autonomous clocks) discussed in our piece refers to the ability to run independently of major temporal programs, such as the cell cycle and/or the circadian clock.

**Figure 1.**
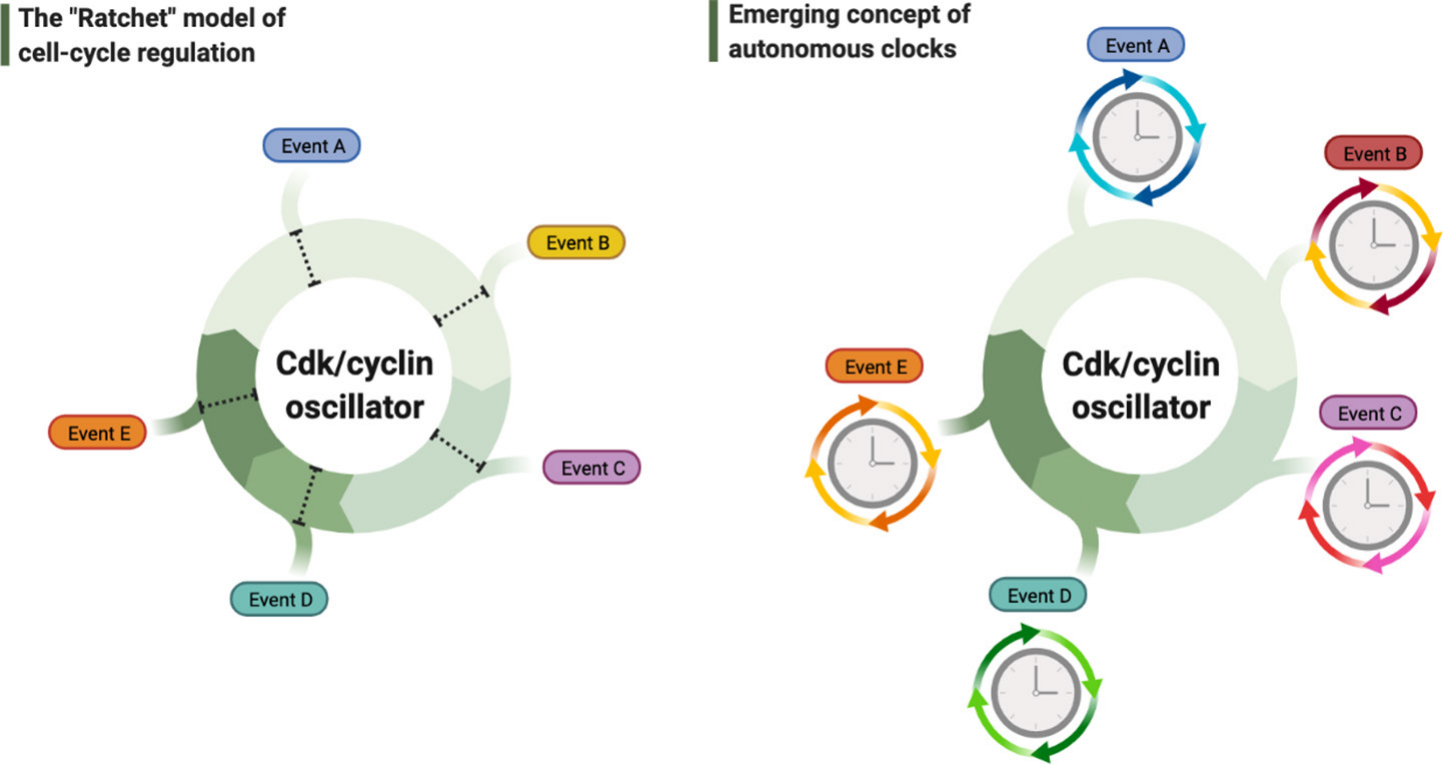
Debated models of the cell cycle. Left diagram depicts the long-standing model of cell cycle regulation where cellular events are triggered directly by the principal cyclin-dependent kinase (Cdk)/cyclin cell division oscillator (CCO). Right diagram describes the emerging concept of autonomous clocks, where the CCO phase-locks the rhythms of otherwise intrinsic mechanisms responsible for timing different cellular events.

Similar to the early adoption of the words ‘autonomous’ and ‘clock’ by the circadian field, in 1953 A. Howard and S.R. Pelc have famously coined the names for different stages of a cell’s division cycle – just as we know and use them today: S-phase, G1, G2, and M-phases (Howard and Pelc, 1953). This nomenclature, which was made solely based on the cycles of chromosome replication, compaction, and segregation, has been unintentionally extrapolated to define the temporal cycle of an entire cell. With the prevailing ‘ratchet’ model on how the principal cyclin-dependent kinase (Cdk)/cyclin cell division oscillator (CCO) may govern these chromosome cycles (Nasmyth, 1996; Stern and Nurse, 1996), such extrapolation has led to a textbook assumption that the CCO must act as a master clock to trigger and order the timing of all events in the cell (Alberts et al., 2002; Figure 1, left panel). This paradigm has recently been under debate as to whether the CCO genuinely defines thresholds to directly trigger all these events, or alternatively, phase-locks a network of local oscillators, each of which may constitute an *autonomous clock* to time the cyclic execution of a specific event (Figure 1, right panel; Lu and Cross, 2010; Morgan, 2010). The ‘phase-locking’ hypothesis was postulated upon the observation that the oscillatory release and uptake of nucleolar Cdc14, a major phosphatase in budding yeast divisions, is normally coupled to cell division cycles, but can continue to operate even when the CCO is halted (Lu and Cross, 2010). However, since perturbing the CCO stops cell divisions, the continuous oscillations of Cdc14 did not appear to have a clear biological role.

Although such functional redundancy in Cdc14 oscillations may have left the original observation unappreciated, evidence that autonomous Cdc14 oscillations can be phase-locked to run at the pace of cell divisions was a major step, particularly to forge a new logic on how such autonomous clocks can be coordinated more generally (Morgan, 2010). This conceptual advance was in contrast to other historical findings on potentially autonomous, periodic biological phenomena, which were considered either molecularly ambiguous or mechanistically epiphenomenal. Ambiguous, because these studies had mostly reported cyclic *output behavior* (e.g., periodic cortex contractions in the eggs of various species, cycles in pH levels, etc.) without clear molecular mechanisms, or functional rationales, as to the underlying biological oscillators (Ciemerych, 1995; Grandin and Charbonneau, 1990; Hara et al., 1980; Shinagawa, 1983; Waksmundzka et al., 1984; Yoneda et al., 1982). Epiphenomenal, because rest of the reported oscillations at *molecular* levels (e.g., p38 MAPK oscillations, rhythms in p53 expression, NF-kB oscillations, etc.) were mostly observed under stress conditions (Bar-Or et al., 2000; Hoffmann et al., 2002; Tomida et al., 2015), and to date, the precise role of their oscillatory expression remains equivocal (Heltberg et al., 2021). Similarly, several other autonomous oscillations that were identified under physiological conditions (e.g., oscillatory activity of K+ channels in mouse embryos, p34 tyrosine phosphorylation cycles in sea urchin embryos, etc.) also remain without definitive functions assigned to their autonomy (Day et al., 1998; Edgecombe et al., 1991). In this piece, we review the latest advances that started to yield novel insights into the molecular mechanisms and potential functions of such autonomous clocks. We present this important concept at different scales and complexities, and postulate original insights, not only for the regulation of cell cycle, but also for broader aspects of biological time control. Our discussions thereby provide a compelling perspective on the broad implications of this emerging phenomenon in physiology and medicine.

### Autonomous clocks at the cellular level: *Discovery of an organelle clock*

The past decade in biological timing research has demonstrated that the pre-mid blastula *Drosophila* embryo is a powerful system to delineate principles of biological time control (Aydogan et al., 2021; Yuan et al., 2016), as major molecules that make up the circadian clock are not expressed until the third instar larvae stage (Yadav et al., 2014), thereby offering an opportunity to study intrinsic temporal programs in the absence of circadian cues.

**Figure 2.**
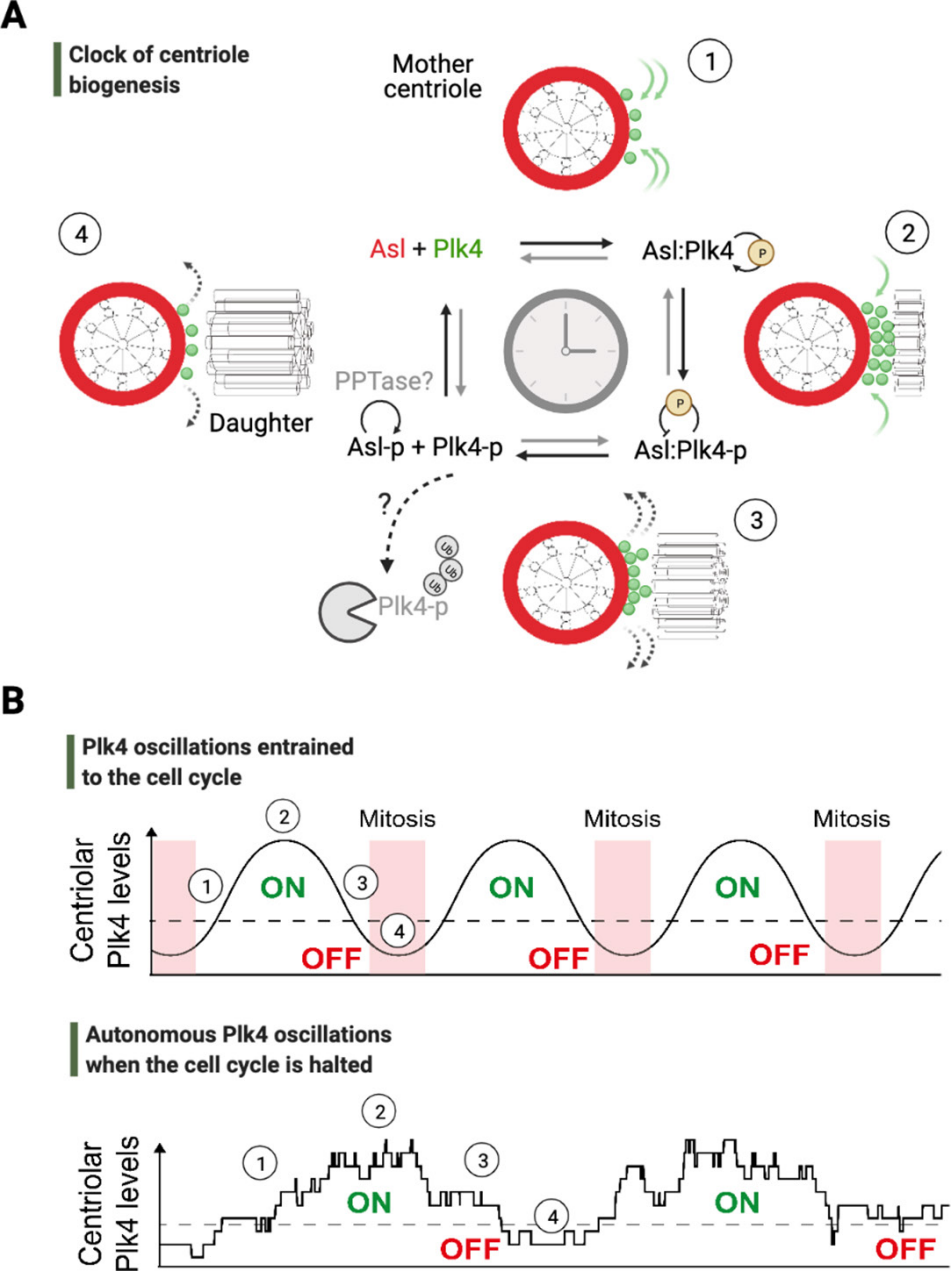
An autonomous clock of centriole biogenesis. (**A**) Cartoon schematic describes proposed steps of the clock of centriole formation (Aydogan et al., 2020). Plk4 (*green*) is recruited to centrioles by its centriolar receptor Asl/Cep152 (*red*) (step 1). Plk4 begins to activate itself by its phosphorylation in trans and trigger daughter centriole formation (step 2). Next, active Plk4 may phosphorylate its receptor to inhibit the binding, leading to Plk4’s departure and/or potential degradation simultaneously to cease daughter centriole assembly (step 3). In order to reset the clock, phosphorylated receptor may be dephosphorylated by a phosphatase to start the process over (step 4). (**B**) Top graph illustrates Plk4 oscillations in entrainment with the cell cycle under wild-type conditions. Here, the Plk4 oscillation data on centrioles can be extracted via taking an average from the entire population of centrioles in fly embryos as the centriole duplication cycle is fully coupled to nuclear cycles. This leads to smoother curves for Plk4 oscillations. Bottom graph depicts Plk4 oscillations in cell-cycle-arrested conditions, where they continue to run and trigger centriole biogenesis autonomously. The duplications of individual centrioles are no longer coupled at the population level, so the individual centrioles duplicate periodically without any obvious synchronization. This leads to noisier Plk4 oscillation curves (as there is much less sampling of the Plk4 signal). Dotted lines indicate the threshold amount of centriolar Plk4 needed to start/stop centriole biogenesis (emphasized by the colored ON/OFF labels). Numbers on both the graphs superimpose relevant steps of the centriole clock model described in (**A**) onto the oscillations in (**B**).

It is in this model system where an organelle ‘clock’ has recently been demonstrated to regulate the timing of centriole formation – an oscillatory mechanism that is normally entrained by the cell cycle progression, but can also run autonomously (Aydogan et al., 2020; Figure 2). Centrioles are cytoskeletal organelles that form centrosomes – the major microtubule organizing centers (MTOCs) that aid the assembly of mitotic spindles in dividing cells (reviewed in Banterle and Gönczy, 2017; Conduit et al., 2015). In most cycling systems, older centrioles (i.e., the mothers) act as a platform where new centrioles (i.e., the daughters) start assembling orthogonally (Breslow and Holland, 2019). Polo-like kinase 4 (Plk4), an enzyme essential for the formation of centrioles, determines the exact site of daughter centriole assembly on the mothers (Kim et al., 2013; Ohta et al., 2014; Sonnen et al., 2012). However, how and when the mothers start forming their daughters, and how these daughters always reach the same size as their mothers, have long remained unknown (Winey and O’Toole, 2014). Recent work has shown that Plk4 localizes to centrioles in an oscillatory manner and appears to determine an effective enzymatic threshold that initiates and times centriole biogenesis (Aydogan et al., 2020; Figure 2A). The adaptive nature of Plk4 oscillations helps to provide homeostasis for centriole growth in fly embryos (Aydogan et al., 2020; Aydogan et al., 2018). Remarkably, Plk4 oscillations persist to run even when the CCO is halted, and they continue to execute centriole formation independently of the cell cycle (Figure 2B). Although such oscillations of Plk4 at the centriole appear to be conserved in mammalian cells (Takao et al., 2019), functionally orthologous mechanisms may regulate the formation of MTOCs in evolutionarily distant species, such as Mps1p kinase that governs the duplication of spindle pole bodies in budding yeast (Blank et al., 2020).

As most cellular processes are intricately coupled to the progression of the cell cycle in dividing systems, what might be the functional significance of Plk4 oscillations independent of the cell cycle? In fact, the self-organizing nature of most mitotic spindles can waive the requirement for a functional centrosome in dividing cells (Basto et al., 2006; Brugués and Needleman, 2014; Heald et al., 1996). In the adult body where most cells are non-dividing, however, centriole biogenesis appears critical due to their function in templating different types of cilia (Basto et al., 2006), purposed for a variety of biological roles, such as the canonical part they play in mechano-chemical signal transduction (Singla and Reiter, 2006). Cilia are an equally essential component of the homeostatic program that regulates organismal health, particularly in respiratory, reproductive, and brain tissues where cells generate streams of vital fluids (Spassky and Meunier, 2017). As these tissues are mostly composed of multi-ciliated cells, tight regulation of centriole number is a chief determinant of cilia abundance. Indeed, recent work on multi-ciliated airway epithelial cells highlights the physiological importance of ciliary abundance and Plk4’s potential role in the homeostatic control of centriole numbers in these non-dividing cells (Nanjundappa et al., 2019). Similarly, another recent study has demonstrated that, in multi-ciliated neuron progenitors, Cdk1 is expressed at low levels that do not trigger mitosis, but is repurposed to *pace* certain steps of centriole biogenesis and cilia formation (Al Jord et al., 2017). Importantly, the same study also demonstrates that such catalytic role of Cdk1 is not necessarily what *drives* centriole biogenesis – evident from cells that continue to produce centrioles (with even higher numbers) when Cdk1 activity is inhibited by pharmaceutical means. These findings collectively implicate a Cdk1-independent mechanism that drives centriole biogenesis in non-dividing cells, while regulating their numbers, at least partially, in a Plk4-dependent way.

Although the molecular basis of such a Cdk1-independent, Plk4-based mechanism for centriole number homeostasis is currently unknown, it is perceivable that the cytoplasmic expression of Plk4 – just as its localization to centrioles – may be controlled in an oscillatory manner to achieve this task. Since cytoplasmic Plk4 levels often correlate with Plk4 levels on centrioles (Aydogan et al., 2020), an oscillatory expression of cytoplasmic Plk4 may guide the frequency and duration of centriolar Plk4 above an effective threshold (to trigger centriole biogenesis): the *frequency* of these integral durations may determine the number of rounds when centrioles can be produced, while the *period* spent above the effective enzymatic threshold may determine the size of centrioles (as depicted in Figure 2). It remains to be determined experimentally whether Plk4 oscillations, or autonomous cycles of another program at the level of proteins and transcripts, can execute centriole biogenesis in these non-dividing tissues.

Beyond the regulation of centriole biogenesis cycles, autonomous Plk4 oscillations could be a paradigm for a general mechanism describing the temporal regulation of organelle biogenesis and cytoskeletal organization: feedback in the levels/activity of key regulatory factors essential for organelle dynamics could precisely time and execute a variety of fundamental processes, from ensuring that organelles grow at the right time and to the appropriate size, to coordinating dramatic changes in their morphology and activity (Bornens, 2021). A unique example to support this paradigm, at least in part, comes from *Cyanidioschyzon merolae*, a unicellular red alga that contains only one chloroplast, which makes it straightforward to study organelle duplication and inheritance more precisely. Intriguingly in these red algae, halting DNA replication stops the cycles of nuclei and cytokinesis, whereas chloroplast duplications continue to occur independently (Itoh et al., 1996). It will be fascinating to see whether any feedback in the levels/activity of proteins that determine the rate of chloroplast divisions, such as PLASTID DIVISION1 and 2 (Okazaki et al., 2009), could time and execute chloroplast divisions. Importantly, another study (Fujiwara et al., 2009) that reports cyclic gene expression patterns during the chloroplast division cycles in *C. merolae* provides other candidate cyclic genes, besides PLASTID DIVISION1 and 2, with which to further investigate the molecular basis of the autonomous chloroplast division cycles.

Finally, if such autonomous clocks operating at subcellular levels were to exist, they would be predicted to display varying enzymatic properties. As such, their response to changes in environmental factors, for example, temperature, would likely differ. A recent study has tested this prediction by examining the syncytial nuclear cycles in early fly embryos under different temperatures (Falahati et al., 2021). In this work, although a large range of changes in temperature (5–22°C) did not alter the order of major mitotic events, under lower temperatures several events of the mitotic entry (such as nuclear envelope breakdown and chromosome condensation) were observed to occur without any detectable rise in the Cdk1 activity or cyclin B levels. This striking finding further supports the existence of autonomous timing mechanisms, which may regulate – among others – even the most quintessential mitotic phenomena that were traditionally considered to be triggered by rising Cdk activities.

### Autonomous clocks at the level of development: *Clues for autonomous timing mechanisms in organismal morphogenesis*

Until recently, studies on developmental timing have helped to reveal only a few autonomous timing mechanisms unequivocally, most notably the segmentation clock (Palmeirim et al., 1997) and the timer that controls the clonal development and differentiation of oligodendrocytes (Temple and Raff, 1986), both of whom can run independently of the cell cycle during development (Gao et al., 1997; Zhang et al., 2008). Further work on potential (cell cycle) autonomous temporal programs that act below mesoscales during organism development, however, remained relatively scarce and uncertain due to insufficient spatiotemporal resolution of imaging techniques, lack of accuracy in targeted gene editing protocols, and the limitations of in situ protein degradation/trapping methods – a problem that in part continues to date.

**Figure 3.**
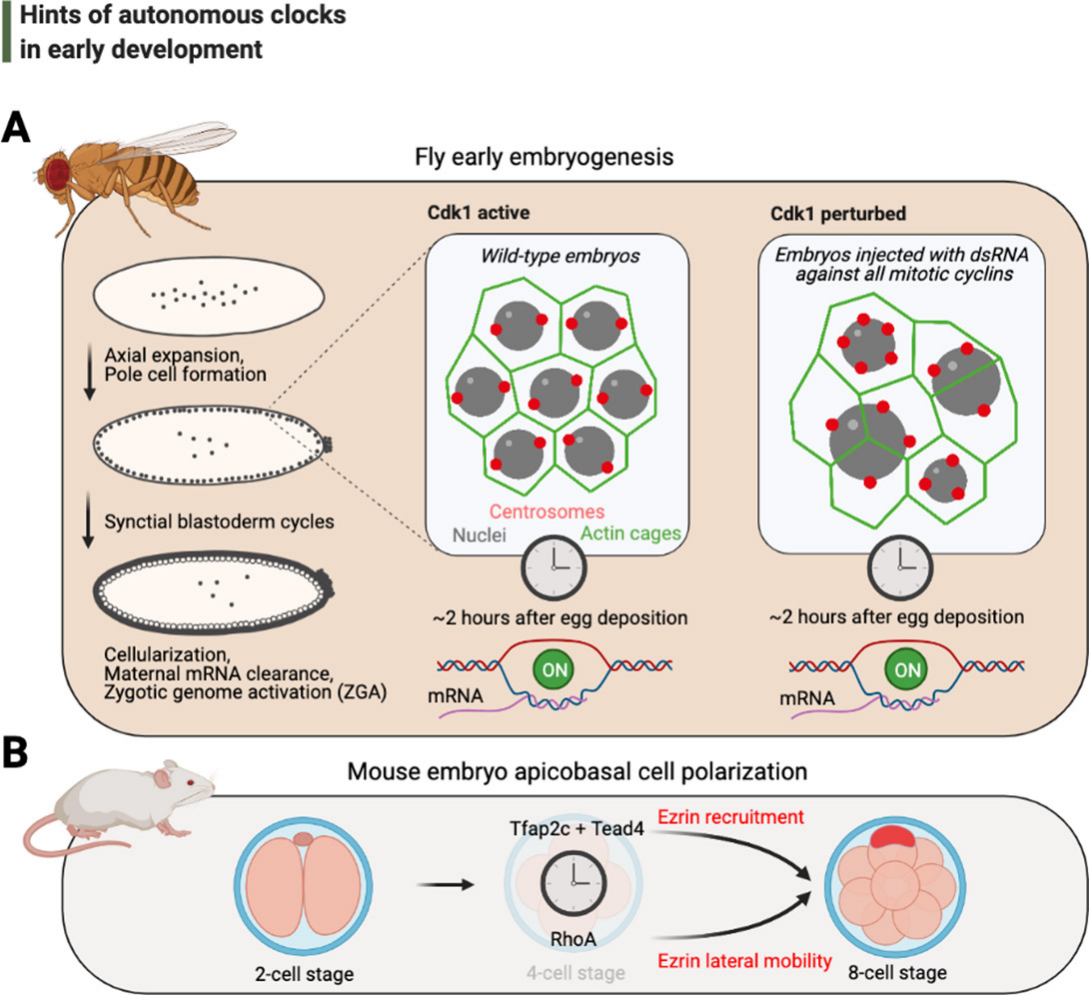
Hints of autonomous clocks in early development. (**A**) Illustrations on the left describe *Drosophila* early embryo development within 2 hr of egg deposition. The two panels on the right describe the cytoskeletal architecture during the syncytial blastoderm cycles in embryos that develop normally (Cdk1 active) or in arrested embryos with 2–3× fewer nuclei (Cdk1 perturbed), which nevertheless cellularize and activate their genome after ~2 hr after egg deposition. (**B**) Cartoons depict recently identified elements of the autonomous clock that is thought to trigger, at least in part, apicobasal cell polarization during mouse embryogenesis. Cdk: cyclin-dependent kinase.

An important and recently debated (Strong et al., 2020; Syed et al., 2021) example of such a potentially autonomous developmental timing mechanism that can run independently of the cell cycle progression is the program that triggers cellularization during mid-blastula transition (MBT) in embryogenesis (Figure 3A). Organismal development unfolds on a tight schedule and in specific sequences that are unlikely to be reproduced by stochasticity. MBT during fly development appears to be no exception: after 14 rounds of synchronous nuclear divisions in the syncytium, thousands of nuclei rapidly become encapsulated by membrane and cellularize within the blastoderm (Farrell and O’Farrell, 2014). Meanwhile, the fly embryo appears to start degrading its maternally provided RNA and simultaneously begins expressing the majority of its zygotic genes (Tadros and Lipshitz, 2009). The major temporal trigger of maternal-to-zygotic transition and its associated events – both in flies and other species – has long been assumed to be the exponential increase in nuclear-to-cytoplasmic (N/C) ratio as a function of cell cycle progression (Edgar et al., 1986; Newport and Kirschner, 1982). With elegant experiments that genetically perturb the progression of cell cycle in fly embryos, recent work suggests the contrary: neither the activation of zygotic genome nor the timing of cellularization is governed by an accurate N/C ratio or cell cycle progression, and both events appear to occur when the nuclear divisions are halted 2–3 rounds in prior (Figure 3A; McCleland and O’Farrell, 2008; Strong et al., 2020). These experiments also reveal a subtle but critical difference between the onset times of cellularization and the zygotic genome activation. Although the slowing of cell cycle progression (in arrested embryos) could explain the premature trigger of zygotic genome activation independently of a set N/C ratio (Strong et al., 2020), the timing of cellularization remains relatively similar to wild-type conditions, eliminating the possibility that a mere slowing in nuclear cycles could elicit the trigger of cellularization. These findings hint at a potential autonomous timing mechanism responsible for the onset of this hallmark morphological event.

What could be the physiological basis of this autonomous timing machinery? Though speculative, a potential mechanism could relate to a series of seminal observations regarding cortex contractions in the unfertilized eggs of various vertebrate and invertebrate species, occurring in periodic cycles that are independent of any cell cycle progression (Ciemerych, 1995; Hara et al., 1980; Shinagawa, 1983; Waksmundzka et al., 1984; Yoneda et al., 1982). Just as cellularization (Schmidt and Grosshans, 2018), the cortex contractions are intimately linked to actomyosin-dependent shrinking and stretching of the embryonal membrane (Chugh and Paluch, 2018), and could potentially count a (right) number of pre-ZGA membrane cleavages that must occur prior to cellularization. Such a maternally supported autonomous timing mechanism may actively inform, or could be monitored by, a set of other molecules that help finalizing cellularization completely. Indeed, previous studies have identified a series of *zygotic* factors that are required for the proper completion of cellularization (both structurally and temporally) (Vastenhouw et al., 2019), but to the best of our knowledge, none of these could elicit an embryonic response that can completely halt the initial *trigger* of cellularization. Therefore, it will be fascinating to see whether these zygotic factors could instead play a major role in coupling cellularization to other hallmarks events during MBT, such as gastrulation.

Although our piece particularly focuses on aspects of timing in de novo cellularization, similar questions on cell cycle autonomous timing can be proposed for other morphogenesis phenomena in early development. Indeed, a recent work has revealed the molecular basis of a long-suspected developmental clock that triggers apicobasal cell polarization independently of cell cycle progression in mice embryos (Zhu et al., 2020; Figure 3B), strongly supporting the possibility of other autonomous timing mechanisms in development and morphogenesis. Such temporal mechanisms could even contribute to differences in the developmental timing of various embryonic structures (i.e., heterochrony), contributing to much of the diversity in body shapes throughout evolution. As yet, much is still unknown, but future investigations into similar questions of time control in development will surely yield fundamental insights – particularly as to whether, and to what extent, other hallmark morphological events could be regulated by such autonomous clocks.

### Autonomous clocks at the systems level*: A sneak peek into novel hints for, and the systematic discovery of, previously unknown autonomous clocks*

Since the 1970s, genetic investigation of circadian clocks has, perhaps single-handedly, dominated the studies of biological time control at the systems level. Evidence that metabolic regulation can be periodic and autonomously regulated (Papagiannakis et al., 2017; Slavov et al., 2011; Benjamin et al., 2005) (i.e., the metabolic cycles) had therefore opened an alternative avenue in this endeavor. Recent research has demonstrated that, similar to the circadian clock (Dunlap and Loros, 2017), metabolic cycles appear to be analogously conserved across species in different branches of the phylogenetic tree; but unlike the circadian clock, the period of metabolic cycles does not appear to be set universally among different organisms (Benjamin et al., 2005; Zhu et al., 2017). Although the function of metabolic cycles may be evident from the point of feeding rhythms, the exact molecular basis of these oscillations remains largely unknown, especially in terms of their interactions with, and potential redundancies when coupled to, either the circadian clock or the cell cycle.

Most top-down studies in the circadian field, such as systems-level analyses concerning diurnal transcriptomes or proteomes, had historically assumed rhythms with peaks every 24 hr and largely disregarded potentially autonomous, non-circadian (i.e., ultradian) rhythms. Pioneering efforts in generating robust statistical methods to study non-circadian periods with low false-discovery rates enabled the discovery of 8 and 12 hr transcriptional rhythms in mice hepatocytes (Cretenet et al., 2010; Hughes et al., 2009). Products of such 8 or 12 hr expressing genes appear to regulate different cellular processes, from protein processing to lipid metabolism, in liver cells (Hughes et al., 2009). How are these non-circadian rhythms of gene expression generated? Are they produced by their own autonomous clocks – just as how the circadian clock can give rise to 24 hr rhythms in gene expression? Or alternatively, do the 8 and 12 hr gene expression simply occur as by-products of metabolic cycling or feeding cues? Several lines of initial evidence suggested that the ~8 and ~12 hr transcriptional rhythms, which were found in cells extracted from living mice, cannot be recapitulated using their cultured counterparts, implying that they might indeed be by-products of systemic signals such as feeding cues (Cretenet et al., 2010; Hughes et al., 2009). Furthermore, with experiments that perturbed several major components in the negative arm of the circadian clock (e.g., Cry1/Cry2), a study demonstrated a significant dampening in the amplitude of 12 hr transcriptional oscillations, indicating that these rhythms must be generated, at least in part, by a circadian clock (and/or a feeding cue)-dependent mechanism (Cretenet et al., 2010). These findings, therefore, favored a working model where the rhythms are driven by systemic, circulating metabolic signals that inform the circadian clock in living organisms, hence cannot be generated in a cell-intrinsic manner ex vivo. As 8 and 12 hr periods are (second and third) subharmonics of 24 hr, theoretical work further substantiated this notion and suggested that such oscillations might be a direct product of circadian clock-regulated activators and repressors that are expressed in an anti-phase fashion (Westermark and Herzel, 2013).

The debates on whether it is an autonomous clock or the core circadian machinery itself that give rise to these oscillations have continued till recently. A major argument in this dispute largely roots from an assumption made by popular oscillation detection algorithms that enforce sinusoid models to determine what *can be* an oscillation (detected from population-level averages), while ignoring the possibility of non-perfect sinusoids and their unique waveforms in single cells (Ballance and Zhu, 2021). It is increasingly appreciated that intrinsic biological noise in individual cells (i.e., stochastic variance in biochemical reactions) can interact with and distort uncoupled intracellular oscillations (Gupta et al., 2016). Consequently, cyclic networks at the level of transcripts, proteins, and/or post-translational modifications are expected to yield imperfect oscillations and irregular waveforms. With analytical methods that enable parameter characterization for superimposed non-perfect sinusoids in single cells (e.g., matrix pencil approaches), recent studies have experimentally re-evaluated how 12 hr rhythms could be generated (Antoulas et al., 2018; Zhu et al., 2017). In stark contrast to previous work, this new heave-ho that brings a methodological twist to the field can recapitulate the 12 hr transcriptional rhythms both in synchronized and unsynchronized hepatic cells – the latter eliminating confounding entrainment effects by the circadian clock (e.g., by tunicamycin incubation) or feeding cues (such as via serum deprivation). Importantly, this stream of work also indicates that the majority of 12 hr rhythmic transcriptome in mouse liver is expressed independently of the circadian clock (Pan et al., 2020; Zhu et al., 2017).

An outstanding question in these studies is to investigate the function of such autonomous rhythms. Although the biological role of 12 hr transcriptional oscillations remains unclear, current evidence – given the nature of the genes involved – points to the spatiotemporal control of central dogma, from gene expression to protein folding. This particularly involves the stress-mediated regulation of gene expression via IRE1α (Cretenet et al., 2010), a stress sensor on the endoplasmic reticulum (ER), and XBP1 (Pan et al., 2020), the transcriptional regulator of unfolded protein response (Walter and Ron, 2011). Pinpointing the exact function of an autonomous clock without proteomic input, however, can be difficult. Emerging technologies that facilitate the study of protein expression in single cells (Budnik et al., 2018; Marx, 2019), as well as protocols that enable systems-level visualization of protein localization and post-translational modifications, will bring equally exciting and new avenues for the study of autonomous clocks. For the latter, the past decade has witnessed considerable progress, particularly as part of the Human Protein Atlas Consortium, in adding time as a fourth dimension to the map of human proteome in single cells (Mahdessian et al., 2021). These efforts will surely be invaluable to not only studying the role(s) of 12 hr transcriptomic clock, but also helping to unravel other potentially intrinsic clocks.

## The logic and potential evolutionary origins of autonomous clocks

### Architecture of complexity and the role of phase-lockers in biological time control

In 1962, Nobel laureate Herbert Simon wrote his pioneering article *The Architecture of Complexity*, postulating the theory that most complex networks (from individual decision-making processes to forming societies) must take the form of hierarchy with unifying properties that are independent of their specific content (Simon, 1962). One such ‘emergent’ property in most complex networks is to acquire numerous nodes that represent subdivisions and to rapidly evolve to interconnect these subunits for robustness. Although Simon was an economist by profession, growing evidence supports the applications of this foundational idea in many aspects of nature – including biology and, more specifically, biological time control.

**Figure 4.**
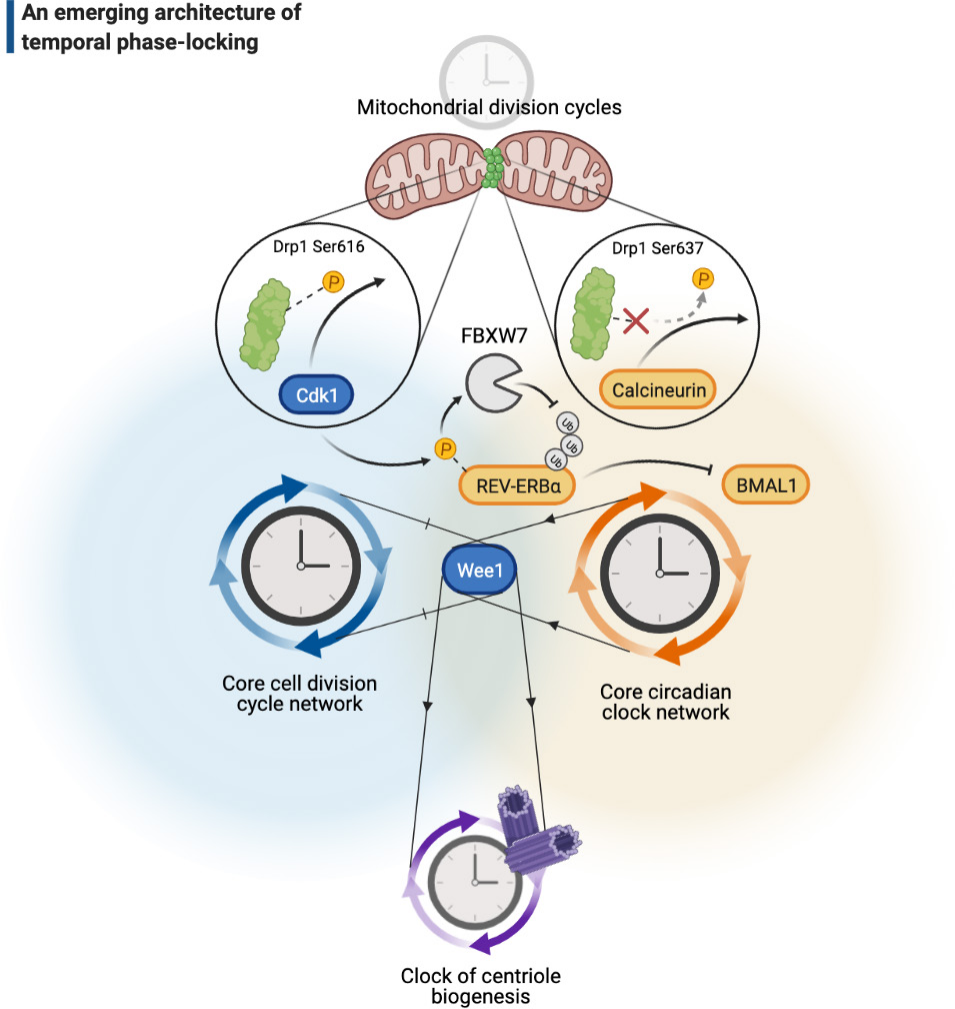
A cellular architecture of phase-locking in biological time control. The simplistic cartoon, guided by recent advances, describes a logic for how intrinsically cyclic biological events (e.g., centriole biogenesis or mitochondrial fission-fusion cycles) can be phase-locked by core components of the cell cycle (e.g., Wee1 or Cdk1) or the circadian clock (e.g., REV-ERBα, BMAL1, or calcineurin) to run at similar paces. Cdk: cyclin-dependent kinase.

**Figure 5.**
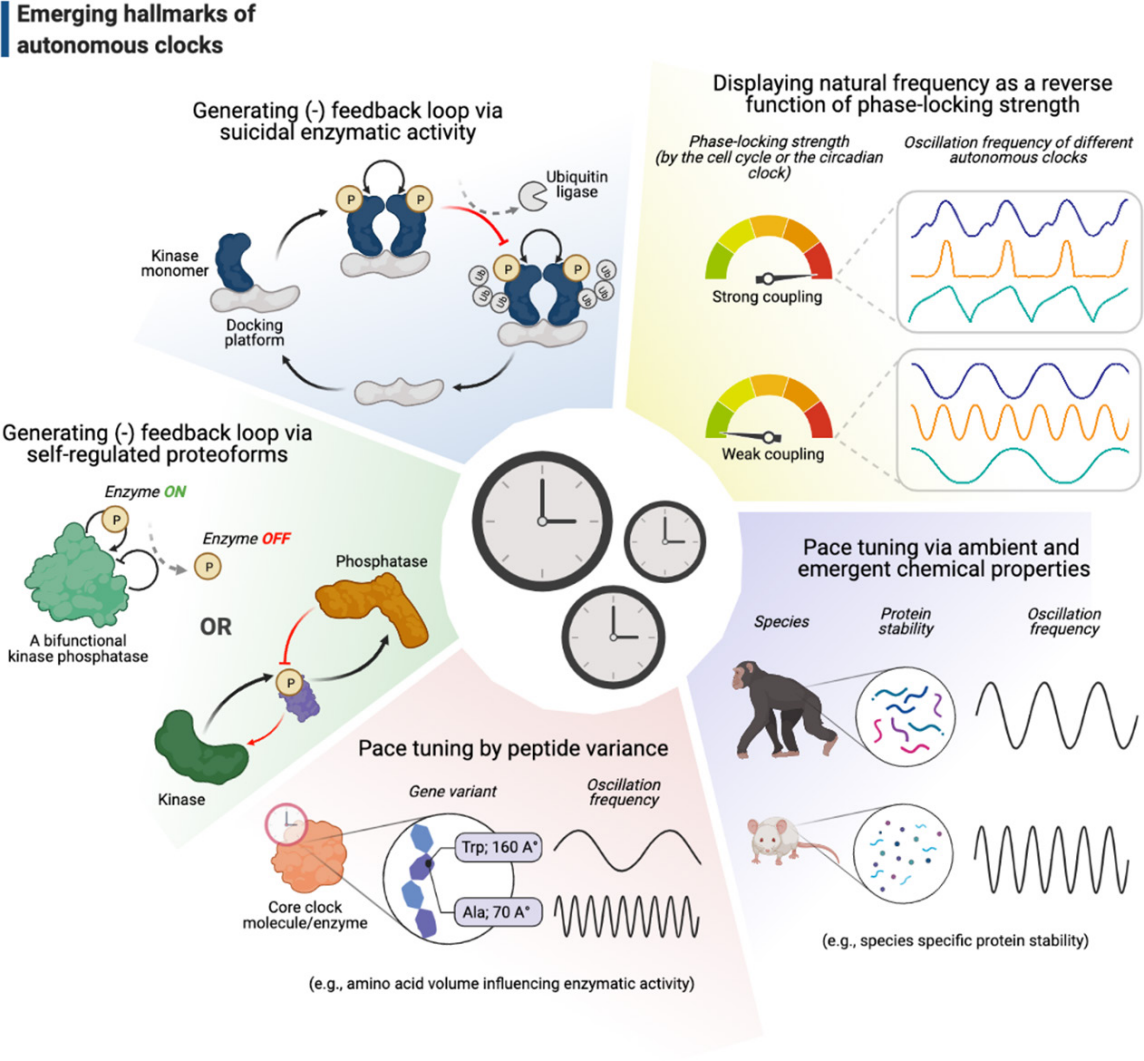
Emerging hallmarks of autonomous clocks. Autonomous clocks may operate via engineering principles that in part display one or more of the five molecular features described in this gallery. Inspired mainly by research in the past decade of biological time control, we suggest these features as emerging hallmarks of autonomous clocks.

Two major networks of temporal regulation in biology, the cell cycle and the circadian clock, have been among the celebrated examples for this concept. The prevailing paradigm of how these temporal networks operate, in particular the cell cycle, holds that a small list of core molecules acts as master temporal regulators to directly trigger all cellular events. But how could a few master molecules achieve their time-keeping function with such little room for redundancy? Namely, if the master molecules are removed from the temporal network, would all subcellular time-keeping activity collapse? Not quite. Emerging evidence highlights the possibility of a network architecture that accommodates many other oscillatory clocks responsible for the execution of different subcellular events (Figure 4). In such a network architecture, the ‘master’ regulators are postulated to act as phase-lockers to unify the periodicity of different events and lock them to constant values (e.g., 1:1 for most cell-cycle-related phenomena; also see the *shaded yellow* slice in Figure 5). For example, although DNA replication and spindle formation are processes that can operate freely in parallel (Hartwell, 1974), they are ‘latched’ to occur once and only once in every cell division. Such phase-locker architecture would predict that either, or both, of these events may occur via self-sustained cycles in the absence of the master regulators. It has been long known that cycles of DNA replication can occur in the absence of nuclear divisions (e.g., the *Gnu* mutants in fly embryos; Freeman et al., 1986), and recent studies on fly development argue that this phenomenon is neither unique to the embryonic tissue nor is a consequence of artificial genetic alteration: multiple rounds of DNA replication not only can occur naturally in the absence of mitosis (Zielke et al., 2011), but also can serve as part of the tissue repair program in various adult tissues (Box et al., 2019; Cohen et al., 2018) – also eliminating the possibility that these autonomous cycles are just an artifact of evolution (i.e., the Darwinian requirement for the utility of intermediate forms; Darwin, 1909).

Such sustained rounds of DNA replication that skew stable cells in favor of polyploidy similarly take up on roles in tissue repair during mammalian development as well (Porrello et al., 2011). However, polyploidy in most proliferating mammalian cells often occur as an unwanted consequence of DNA damage or due to defects in chromosome segregation (Chow and Poon, 2010) and, unlike in fly endocycling cells (Zhang et al., 2014a), leads to p53-mediated cell death. How could terminally differentiated mammalian cells tolerate a mismatch between their DNA content and cell size, whereas their proliferating progenitors cannot? One idea that can explain this discrepancy is that, although majority of the mammalian proliferating cells maintain high levels of p53 expression, cells that undergo terminal differentiation may experience a significant decline in p53 levels (Tedeschi and Di Giovanni, 2009). But for now, the ideas that can explain this difference between proliferating and stable cells are just that: ideas.

### Mechanisms of biological phase-lockers

In view of such complexity, it is no surprise that the cell cycle network contains circuits that are capable, on their own, to produce autonomous cycles. Given the late divergence of Cdk1 in the evolution of the metazoan cell cycle (Krylov et al., 2003; Nasmyth, 1995), the prominence of such self-sustained clocks and the type of autonomy they display to run independently of the cell cycle network appears to be more ubiquitous than previously thought. But how could only a handful of core molecules in the cell cycle phase-lock to organize such autonomous cycles?

Although there are a number of ways and conditions that help phase-locking two or more biological cycles (Heltberg et al., 2021), post-translational regulation of protein stability has emerged as a prominent target for an oscillator to couple another one. In this realm, the enzymatic ability to induce protein modifications (e.g., phosphorylation) with varying levels of potency and efficacy (Holt et al., 2009; Swaffer et al., 2018; Swaffer et al., 2016; Ubersax et al., 2003) may elucidate how cell-cycle-driven proteolytic activities could couple cycles of cell divisions to peripheral biological cycles that are otherwise autonomous. The clock of centriole biogenesis in fly embryos, for example, appears to be phase-locked to the cell cycle by a molecular mechanism that is – expectedly – in mitosis (Aydogan et al., 2020). As the embryo progress up to its 13th round of nuclear division before the zygotic genome activation, however, the centriole clock acquires an additional mechanism that strengthens its phase-locking to the cell cycle by an extra step of coupling in interphase. It is well known for this stage of embryogenesis that Wee1, a master kinase of cell cycle regulation, dramatically slows the cell cycle (Stumpff et al., 2004). In line with this, embryos that are deficient of Wee1 lose much of their robustness in coupling the centriole cycle in interphase (Aydogan et al., 2020; Figure 4). This example clearly illustrates how different core components of the cell cycle network that normally counteract proteolytic activity can also help phase-locking an autonomous clock – in a manner that is dependent on both the stage of cell cycle and organismal development.

The exact molecular mechanisms of biological phase-locking are still largely unknown; however, various patterns in enzyme-substrate modifications may provide useful insights for future research. For instance, cell-cycle-dependent post-translational modifications are thought to be modulated either stably via high specificity on a small number of select residues with motifs that lend regular secondary structures, or promiscuously (in a dynamic manner) on intrinsically disordered chunks of peptides (Gunawardena, 2005; Tyanova et al., 2013). We speculate that such alternating modes of protein modifications may correspond to differential regulation of phase-locking (in both space and time) during the cell cycle. While stable number and sites of modifications may allow for programmed phase-locking events with individual proteins more locally (such as phosphorylating a protein that is essential to couple, say, only one round of cytokinesis to every mitosis), bursts of futile modifications on disordered peptide chunks may promote more rapid synchronization at a global level, particularly in response to homeostatic events such as growth signals and metabolic cues. Computational and systems-level studies that have examined the bulk phosphorylation events in circadian (Upadhyay et al., 2020) and cell cycle regulation (Swaffer et al., 2016; Tyanova et al., 2013) draw similar conclusions to the latter part of our speculation. With a few exceptions in vivo (Deshaies and Ferrell, 2001; Nash et al., 2001), the former idea is pending to be vigorously tested in vitro for residue specificities.

Is the activity of phase-locking molecules exclusive to certain substrates in a specific temporal network, or could they be used more widely in different networks of time control? Even though potential answers to these questions can be confounded by a variety of factors related to differentiating levels of enzyme activity, or of substrate specificity, existing evidence suggests the intriguing possibility that such molecules may not be fully dedicated to their original context and might be more ‘universal’ (Figure 4). For example, a number of classic and recent studies have strongly indicated that rhythms of the circadian clock and the cell cycle are phase-locked to run at a constant ratio (1:1) (Bieler et al., 2014; Yan and Goldbeter, 2019) – the circadian expression and activity of Wee1 appears to couple the cell cycle to the circadian clock (Matsuo et al., 2003), while the Cdk1 activity couples the circadian clock to the cell cycle via phosphorylating REV-ERBα (Zhao et al., 2016; i.e., phase-locking the phase-lockers; Figure 4). Therefore, biological phase-lockers may be employed in ways that are not mutually exclusive between different temporal networks. On the other hand, the substrates may not be mutually exclusive either. For example, the activity of mitochondrial fission enzyme Drp1 can be coupled to both the cell cycle (Taguchi et al., 2007) and the circadian clock (Schmitt et al., 2018). While Drp1 phosphorylation (on Ser616) by Cdk1 leads to its activation (Taguchi et al., 2007), dephosphorylation (on Ser637) by calcineurin (Cereghetti et al., 2008), a circadian-regulated phosphatase, can serve for the same function as well (Figure 4).

In our piece, we highlight specific examples of phase-locking with 1:1 ratio as they appear to be the most common form of coupling in nature – for every one period (*p*) that is completed for a biological cycle implies a single complete period (*q*) for the other. There are, however, other ratios (*p:q*) of coupling that arise in biology. A unique example is the 1:2 phase-locking between the maturation of daughter centrioles (to become full-fledged centrosomes) and the cell cycle (Palazzo et al., 1999). Each daughter centriole takes two full cell cycles to complete their maturation to serve as centrosomes and organize spindle formation. A difference in this ratio can bring pathological ramifications: either as monopolar spindles for locking ratios < 1:2 (such as 1:3) or as multipolar spindles for locking ratios > 1:2 (such as 1:1), leading to aneuploidy (Compton, 2011; Godinho and Pellman, 2014; Hinchcliffe, 2011). For centrosome maturation and its coupling to every other cell cycle, 1:2 locking ratio is conserved stably through evolution, presumably for fitness purposes, and applies to many – if not all – dividing cells. But in general terms, when two such biological cycles are phase-locked, does their locking ratio *p:q* have to be the same for every single coupling event, say, across a population of cultured cells? Or, could different phase-locking ratios mutually exist for two interacting biological cycles, even under what appears to be similar environmental/experimental conditions?

The short answer is a surprising *yes*. For instance, our discussion on the coupling between circadian clock and cell cycle has focused mostly on their 1:1 phase-locking, yet a recent study has experimentally demonstrated that both 1:1 and 1:2 phase-locking ratios can mutually exist even among the same population of cells (Droin et al., 2019). In other words, cells can couple every ~24 hr of the circadian turn to either one (1:1) or half (1:2) a division cycle. As unexpected as this may be to the biologist, it is no surprise for the theoretical understanding of conditions under which phase-locking can occur. In a parameter space where axes represent the locking ratio *p:q* (of two interacting biological cycles) and the locking strength, various phases of coupling can emerge for different *p:q* values. This is because increasing the locking strength often increases the span of coupling for most plausible *p:q* ratios (Heltberg et al., 2021), leading to potential overlaps between different *p:q* values (ergo the observation that they can coexist in the same cell population). This positive relationship between the locking strength and ratios, famously called the ‘Arnold Tongue,’ is a useful graphical tool to assess the conditions under which phase-locking can emerge. Although an extended discussion of Arnold Tongues is beyond the scope of our piece, several other cases relating to phase-locking (e.g., mode hopping, period-doubling, chaos dynamics) can be qualitatively inferred from Arnold Tongues to explore underlying biological mechanisms. For further details, we refer our reader to a recent piece (Heltberg et al., 2021) that summarizes this concept in ways that are fully accessible to the biologist.

## Molecular determinants and roles of autonomous clocks

### Emerging hallmarks of autonomous clock mechanisms

Gene regulatory mechanisms proposed by Jacob and Monod, 1961, involving activators and suppressors that feedback on message synthesis via gene expression, have long inspired a variety of concepts in systems biology, particularly including the pioneering theoretical work by Brian Goodwin on how clocks in biology may operate (Goodwin, 1963). The mechanism of circadian clocks is a prime example, where complicated maps of transcription-translation feedback loops (TTFLs) have become an indispensable part of this field (reviewed in Takahashi, 2017). Although TTFLs appear important in maintaining the robustness of, and sustain prolonged rhythms for, circadian clocks (Putker et al., 2021), several studies have strongly challenged the *necessity* of TTFLs to run the clocks themselves (Lipton et al., 2015; Nakajima et al., 2005; Tomita et al., 2005), including the seminal observation of circadian redox rhythms in enucleated mature red blood cells (O’Neill and Reddy, 2011). The unifying feature of these studies is their proposal of alternative mechanisms at the level of proteins, such as the use of autoregulatory post-translational modifications to generate self-sustained feedback loops (Figure 5, the *shaded green* and *blue* slices). For instance, the bifunctional kinase/phosphatase nature of KaiC ATPase in the cyanobacterial circadian clock is the only necessary and sufficient feature that enables the Kai ABC oscillator to run autonomously (Nakajima et al., 2005; Terauchi et al., 2007). Similarly, the autokinase activity of Plk4 might contribute to the clock of centriole biogenesis to run independently of cell cycle progression: Plk4’s recruitment to centrioles may promote both the inhibitory phosphorylation of its receptor (Aydogan et al., 2020; Boese et al., 2018) and its autophosphorylation in trans that triggers its ubiquitylation (Cunha-Ferreira et al., 2013; Guderian et al., 2010; Holland et al., 2010; Rogers et al., 2009). These might lead to Plk4’s departure from centrioles and/or its degradation, respectively (Figures 2A and 5). Although Plk4’s departure from its receptor may not be mutually exclusive with its degradation in parallel, it is tempting to postulate that such self-triggered ‘suicidal’ degradation of Plk4 may contribute to its function as a homeostatic clock during centriole biogenesis (Aydogan et al., 2018).

These advances indicate that other such autoregulatory enzymes (Lu and Hunter, 2009) (endowed with the ability to both promote and impede their own activity) could act similarly to form self-sustained feedback loops and regulate biological timing at subcellular levels. An intriguing example is protein kinase C (PKC) – a suicide enzyme whose autophosphorylation facilitates both its activation and degradation (Feng and Hannun, 1998; Gould and Newton, 2008). PKC activity has been demonstrated to act as a master switch to trigger lysosome biogenesis and to do this independently of mTOR-mediated signaling for cellular growth (Li et al., 2016). More surprisingly, although PKC-mediated biogenesis of lysosomes is *coupled* to the cell cycle by CDK4/6, halting cell cycle progression (either by the inhibition of CDK4/6 or mitotic Cdk1/2) does not prevent further lysosome biogenesis (Yin et al., 2020). Whether PKC can act as an autonomous subcellular ‘clock’ to initiate and time lysosome biogenesis, or other such autoregulatory enzymes to do similar time-keeping tasks for different subcellular events, remain to be elucidated.

How could autonomous clocks set their pace? Artificially induced, or naturally occurring, gene variants that result in a dramatically long or short oscillation period may offer useful molecular insights (Takahashi, 2004). In terms of circadian clocks, this approach has seldom revealed features that can pinpoint exact molecular mechanisms as the clockworks are fairly intertwined in most eukaryotes. A recent study, however, was able to break open the merits of this approach by following varying phenotypes of circadian clock period in cyanobacteria (Ito-Miwa et al., 2020). Strikingly, the study has demonstrated that the volume of a single residue (402) on KaiC dodecamers induces a strong negative response on the overall period of KaiABC oscillator – the bulkier the residue, the shorter the circadian period (e.g., while a Tryptophan variant in residue 402 induces <24 hr period, alanine results in a period of 5 days; Ito-Miwa et al., 2020; Figure 5, the *shaded orange* slice). Although this study finds a strong correlation between the variant phenotypes and KaiC ATP-ase activity to explain the vast period differences, what still remains inconclusive is whether this residue somehow directly influences the ATP-ase activity via an allosteric mechanism at the interface of two KaiC hexamers or indirectly by disrupting the formation of KaiC dodecamers, hence perturbing the clock synchronization.

Finally, we must caution that the tuning of autonomous clocks may not solely depend on features that are attributed to specific base or peptide sequences, especially if the mechanisms that set the oscillation period depend on emergent chemical properties, such as protein stability (Figure 5, the *shaded purple* slice). For example, the period of p53 oscillations – a response to DNA damage conserved across mammals – can vary dramatically between different species due to differing rates of p53 degradation (Stewart-Ornstein and Lahav, 2017). While the low stability of p53 leads to rapid oscillations in rodents (~3 hr), the rhythms appear two times slower in primates (~6 hr). Although the exact function of this period difference remains unclear, recent independent work has shown that the level of protein stability between these two species varies not only for p53, but at a global level (Matsuda et al., 2020; Rayon et al., 2020). Strikingly, one of these studies (Matsuda et al., 2020) has further demonstrated that, just as the p53 oscillations, the segmentation clock in mice runs (~3 hr) twice as fast as the one in humans (~6 hr). This emphasizes the importance of monitoring systems-level emergent properties that can indiscriminately influence biological timing in a way that is clearly independent of the individual molecular components that make up the intrinsic clocks themselves.

### Autonomous clocks in health and disease

Maintenance of cellular events in non-dividing cells, particularly including the dynamics of their organelles and cytoskeleton, has long been considered a ‘simple house-keeping task’ (Lowe and Barr, 2007). Latest discoveries in autonomous clocks, from the biogenesis of an organelle to complicated self-organization events that shape morphogenesis in development, argue that such a ‘static’ view of the non-dividing cell is no longer valid. This emerging picture of active regulation in non-dividing systems is also supported by studies that demonstrate dramatic reshaping of organelle morphology (Buck et al., 2016; Iwata et al., 2020; Kasahara et al., 2013; Mitra et al., 2012) and cytoskeletal structure (Pongkitwitoon et al., 2016; Treiser et al., 2010; Yourek et al., 2007) as part of cell fate determination.

An important contributor to organelle dynamics, among numerous others, is the nuclear transcription programs that provide messages to produce essential building blocks in the cell. Although there are multiple modes of controlling transcription in time, which predominantly include stochastic expression of genes that maintain cellular heterogeneity (Kaern et al., 2005; Raj and van Oudenaarden, 2008), cyclic regulation of transcription in bulk, and its potential biological roles, has also attracted significant attention (Klevecz et al., 2004; Lee et al., 2002; Simon et al., 2001) and led to a number of recent questions: Why should cells express a portion of their genomes periodically? Could such periodic expression be achieved independently of major temporal programs, such as the cell cycle or the circadian clock? If so, how?

Studies of yeast metabolic cycles (YMCs; Benjamin and McKnight, 2007) pioneered the investigations on how cyclic expression of genes can possibly shape organelle dynamics. For example, a landmark study by McKnight and Tu (Benjamin et al., 2005) revealed periodic expression in three superclusters of budding yeast genes that appears to temporally *compartmentalize* the YMC: one oxidative and two functionally distinct reductive stages. Most intriguingly, the reductive stages bring up dramatic changes in organelle biology, first by promoting the synthesis of products needed for mitochondrial biogenesis – presumably to replenish these organelles after their use in the anabolic stage, then triggering vacuole biogenesis to promote the degradation of cellular waste as part of the catabolic process. It is important to recognize that the YMCs occur at a period of 4–5 hr, which is ~3× longer than a regular cell cycle in budding yeast and ~5× shorter than the circadian rhythms; highly suggestive of their temporal autonomy.

Autonomous transcription cycles may not be exclusive to the genes expressed during the YMC, however. Another study in budding yeast has provocatively demonstrated that, although a large portion of the genome is transcribed cyclically during the cell division cycle, a significant fraction (~70%) of these genes can continue to express periodically even in the absence of a robust cell cycle progression (Orlando et al., 2008). This striking finding has rightly inspired a number of more recent work (Cho et al., 2019; Cho et al., 2017; Rahi et al., 2016) to validate these findings, and although there does not seem to be a strict consensus, it is somehow clear that the periodic transcription in arrested cells may not be as *global* as it was originally postulated. Importantly, however, there appear to be some genes that can display periodic expression in the absence of a robust cell cycle oscillator. One of these, *Sic1*, offers important insights into a potential function for such an autonomous expression cycle: Sic1 provides a mechanism that promotes nuclear divisions when levels of mitotic cyclins are low to trigger them (Rahi et al., 2016). This demonstrates a fail-safe mechanism that cells can use to sustain robustness of the cell cycle or a means to reactive the progression of the cell cycle in states of quiescence.

Such examples of cyclic transcription in yeast provide important functional clues for oscillatory genome expression independently of the circadian clock and/or the cell cycle. Although we single out potential roles that appear exclusive to these autonomous transcription cycles, whether they may share similar biological roles with transcriptional cycles governed *directly* by the cell cycle or the circadian clock remains unknown. Except, an earlier study has postulated such an overarching, ‘energy-conservation’ function by drawing parallels between the autonomous YMCs and the circadian expressing transcriptomes from flies to humans (Wang et al., 2015). By theoretically assessing the metabolic costs for cyclic and non-cyclic transcripts within each species, the authors have predicted that cyclic expressing genes must have a greater energetic price for the cell. Yeast chemostat experiments in this work indicated that, under high glucose conditions, significantly more transcripts begin to display oscillatory expression. Since most of the newly cycling transcripts are enriched for gene annotations related to metabolic functions, the authors postulate that oscillations in gene expression may help satisfy the homeostatic demands of high glucose conditions: increasing gene expression for certain time windows could meet the demand when needed, while decreasing the expression for alternating periods could avoid futile production. As such, the mean expression may remain relatively constant and ‘average out’ the energetic cost.

As elegant as this idea may be, alternative interpretations can be reasoned from the same observations. Previous in vitro evolution experiments have demonstrated that cyclic behavior in yeast can arise as an emergent property of evolving (mutated) transcripts without any cyclic environmental or metabolic cues (Wildenberg and Murray, 2014). If oscillatory gene expression is such an emergent property, especially if one that is costly for the cell, it may be actively suppressed for cells that grow in low glucose conditions. Once high levels of glucose are provided, the restriction on this emergent property might be released, allowing genes to express in oscillations. Therefore, the exciting ‘energy-conservation’ idea for oscillating transcripts (Wang et al., 2015) remains to be tested more directly for causality.

Our discussion on the potential roles of autonomous clocks has so far largely revolved around a variety of molecular processes under healthy physiology, and by definition, when these processes go corrupt in the aging body, they can lead to degenerative illnesses. Some diseases, on the other hand, occur as a result of external factors, such as infection. Among these, malaria has been particularly intriguing as, upon *Plasmodium* infection, its symptoms tend to occur in a cyclic manner (i.e., every ~24 or 48 hr; Hawking et al., 1968). This periodicity phenomenon has long been considered a consequence of host-driven circadian responses. In contrast, a pair of recent studies have strikingly demonstrated that, although the intraerythrocytic developmental cycle of *Plasmodium* parasites can be *entrained* by host-provided extrinsic cues (such as the host circadian clock or feeding rhythms), it is likely *run* by an autonomous clock that regulates their transcriptional cycles – periodic gene expression events that operate with features that resemble, but nevertheless appear to function independently of, the cell cycle and/or an innate 24 hr circadian clock (Rijo-Ferreira et al., 2020; Smith et al., 2020).

Upon infection of the host systems, most pathogenic organisms form ‘inclusions’ to create a membrane-bound, organelle-like compartment to protect themselves from host-driven autophagy or exocytosis responses (Moore and Ouellette, 2014). If the relationship with the host proves mutually viable over evolution, an endosymbiotic interaction may emerge, as had happened with the incorporation of mitochondria and chloroplast into the eukaryotic cytoplasm (Sagan, 1967). During endosymbiosis, the movement of proliferation-related genes from guest to host genome (i.e., horizontal gene transfer) appears to have helped hosts to take over the control of organelle-like compartment divisions (Husnik and McCutcheon, 2018). Such gene transfer in *Plasmodium* infection is difficult to imagine as they infect and form inclusions in mature red blood cells lacking nuclei. However, many other parasites do form inclusions: beyond their well-known capability to hijack the proliferation and enzymology of host organelles, these inclusions have recently been conceptualized as de novo parasitic organelles that may regulate their own cycles of development (Moore and Ouellette, 2014). Although how these cycles are temporally regulated is currently unknown, they may shed further light on the evolution of other autonomous clocks in the cell.

### What’s past is prologue: The future of autonomous clocks

Instead of purely speculating on the future of autonomous clocks, we would like to take an active stance to describe the current challenges in their identification so as to highlight potential experimental approaches that may help to address some of the major outstanding questions in biological time control (summarized in Box 1). By no means are our thoughts exhaustive, but they are potentially useful insights to follow for future discoveries.

Box 1. **Outstanding questions on the emerging concept of autonomous clocks.**

1. Just as centrioles, could the biogenesis and/or dynamics of other organelles be regulated by autonomous clocks? In proliferating cells, these clocks would be strongly entrained by the principal Cdk/cyclin cell division oscillator (CCO), but when/if the CCO is inactivated or perturbed, they may operate independently.
2. What would be the function of potential autonomous clocks? Are they used as fail-safe mechanisms during states of quiescence in proliferating cells (i.e., standby time machines when the CCO is damaged)? Could they be used as pacemakers in non-dividing cells to define cell fate or terminal identity (e.g., an endoplasmic reticulum expansion clock in secretory cells, or a vacuole biogenesis clock in adipocytes)?
3. Do autonomous clocks act locally in subcellular settings? Could they be synchronized across the cell with intrinsic factors other than the CCO-provided cues (e.g., membrane contact sites or waves of other factors)? Could they be synchronized across a tissue with cell-to-cell interaction mechanisms (e.g., nanotubes or exocytosis)?
4. How could an autonomous clock evolve?
  - Did different cellular events evolve to control time with their autonomous clocks and become progressively coupled to a common cell cycle?
  - Was the primitive version of eukaryotic cell cycle a network of roughly coupled autonomous clocks? Did the CCO evolve to become a master regulator for this network?
  - Are autonomous clocks just an intermediate form of cellular timing in evolution? Or are they employed actively, even in species where the CCO or the circadian clock imposes a strong degree of phase-locking?
  - Did autonomous clocks require extrinsic cues during their evolution (e.g., light, feeding, ionic fluxes, etc.)?
5. Do autonomous clocks operate with common physical or chemical principles?
  - Could suicidal enzyme activity be a general design principle of autonomous clocks (i.e., enzyme degradation occurs as a function of increase in activity)?
  - Are there other molecular signatures of autonomous clocks, such as DNA sequences or peptide motifs?
6. Apart from the segmentation clock, as well as hints that suggest potential clocks for cellularization or apicobasal cell polarity, could other hallmark developmental events be regulated by their own clocks?
  - Do potential developmental clocks crosstalk?
7. At the systems level, similar to the autonomous 12 hr metabolic cycles in mammalians, there appears to be a third subharmonic of the circadian rhythms (~7–8 hr oscillations in gene expression). Are 8 hr oscillations independent of the cell cycle or the circadian clock? If so, what is their function?

**Figure 6.**
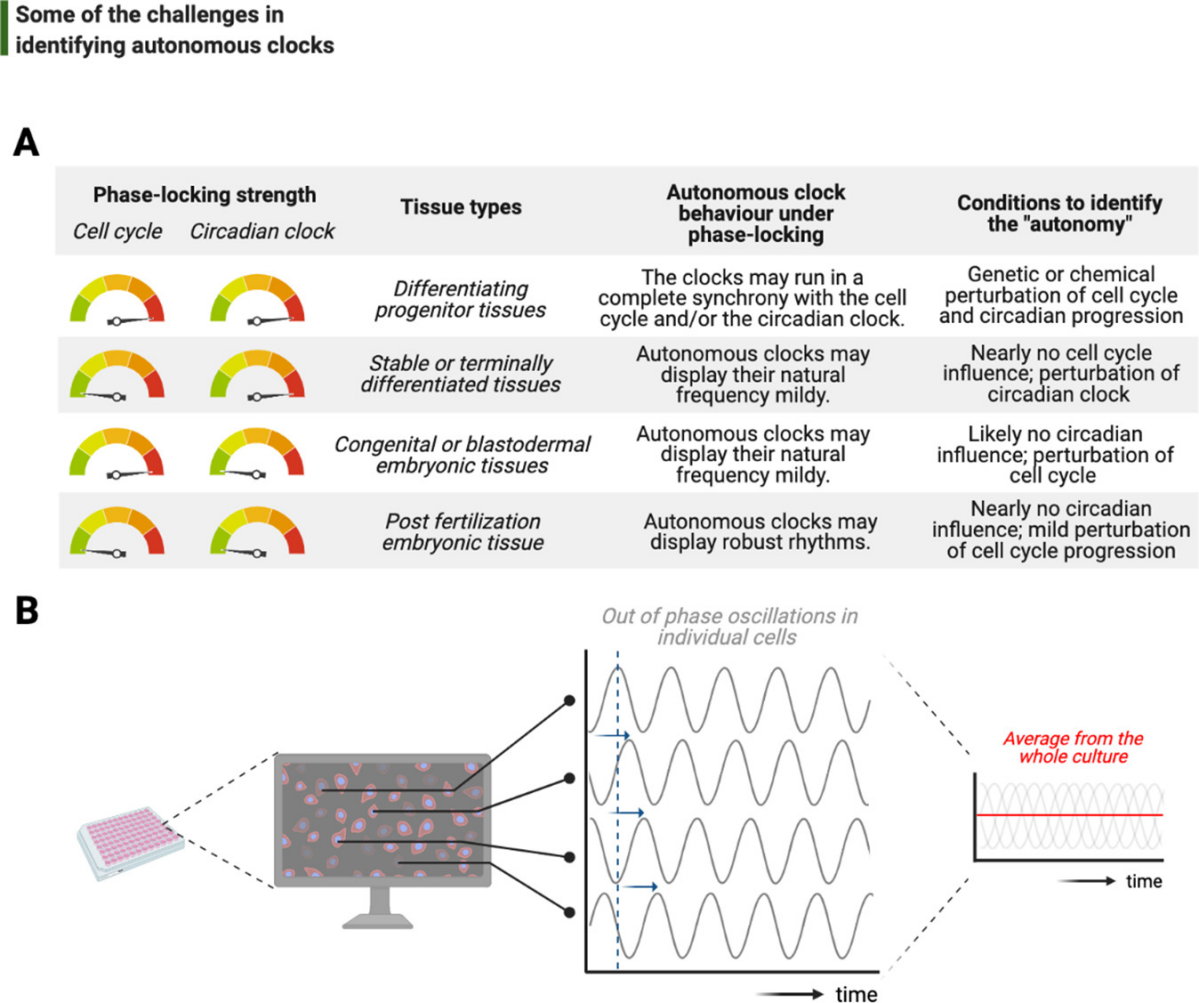
Challenges associated with the study of autonomous clocks. (**A**) In most metazoans, biological events are usually phase-locked to run at the pace of the cell cycle and/or the circadian clock. The table provides examples for varying combinations of phase-locking strengths (when the strength is high, the gauge indicator points to *red*, or vice versa for *green*) and how these combinations are employed by different types of tissues during the development (first and second columns, respectively). Depending on the tissue type, autonomous clocks may display their natural frequency more easily in some (e.g., embryonic tissues) than others (e.g., proliferating progenitor tissues) (third column). We suggest experimental conditions in which novel autonomous clocks could be identified with a higher degree of confidence in interpreting their ‘autonomy’ (fourth column). (**B**) The flow chart illustrates a hypothetical high-throughput screen to identify novel autonomous clocks. If a prospective clock is not synchronized between cells and displays phase shift from one cell to the other (indicated by *sliding blue* arrows on the graph) due to the lack of an entrainment cue, then simply taking an average of rhythms in all cells may lead to a flat line. This will inevitably result in false conclusions and missed opportunities.

One of the foremost and complicated decisions that goes into the study of intrinsic rhythms is the choice of model system, from the type of cell to species (Figure 6A). In a previous section, we discussed two major biological phase-lockers, the cell cycle and the circadian clock, that can entrain the rhythms of otherwise autonomous clocks. The degree of phase-locking by these temporal programs plays a crucial part in identifying autonomous clocks: the stronger the phase-locking, the more likely that potentially intrinsic rhythms can be rendered redundant over evolution, hence are more difficult to unravel (Lu and Cross, 2010; Morgan, 2010). A brief comparison in the number of circadian regulated transcripts between land and sea species illustrates the strength of circadian phase-locking in the former: while almost 50% of all mouse genes show circadian expression in at least one organ (Zhang et al., 2014b), this fraction is only 10% in *Bathymodiolus azoricus* in situ in deep sea (Mat et al., 2020).

Conceptual parallels on temporal coupling can be drawn with the ‘checkpoints’ of the cell cycle regulation: the more complex the multicellularity traits are, the stronger the checkpoints appear. For instance, mammalian cells cannot tolerate prolonged arrests in mitosis and activate the p53-dependent apoptotic pathway soon after their arrest (Chen, 2016). The undesired cell death in arrest conditions clearly makes it an onerous task to study potentially intrinsic cycles in this system. Similarly, the number and type of Cdks appear to change based on multicellularity traits. While yeast regulates the entirety of its cyclins with a single Cdk, the number of different Cdks in mammalians reaches double digits (reviewed in Malumbres, 2014; albeit not all directly related to the core regulatory mechanisms of cell cycle progression). Fittingly, it is yeast that had been a hotbed of phenomena that occur periodically in arrested cells, notably including free-running budding cycles (Haase and Reed, 1999) and the rate of CO_2_ production (Novak and Mitchison, 1986). In most of these cases, although the baseline clocks are not known, the ‘output phenotypes’ are a salient indication of underlying timing mechanisms that deserve further attention.

A recent example for such an *output phenotype* in mammalians is the potentially autonomous oscillation of growth rate in cultured human cells, revealed by quantitative phase microscopy (Liu et al., 2020). These oscillations are indeed autonomous of the cell cycle as they continue to run in S-phase-arrested cultures. However, the oscillations are ~4 hr on average, namely a sixth subharmonic of the circadian period. Intriguingly, this work shows that rapamycin partially dictates the oscillation period, indicating potential involvement of mTOR (an important regulator of cell growth) in generating these oscillations. Supporting our hypothesis that the free-running circadian clock might be a significant confounding factor, mTOR activity has recently been demonstrated as a target of Per2 (Wu et al., 2019), a core circadian clock protein. Nevertheless, such oscillations in mammalian cell growth are striking, but require future investigation to address their biological function and relevance, particularly in terms of the complex regulation between cell growth, division, and size control.

Finally, although recent bottom-up efforts have provided tremendous molecular insights into how autonomous clocks may work, their identification has been limited to the interest of individual investigators. In order to scale up the discovery of potentially other intrinsic timing mechanisms, *reliable* use of the existing top-down approaches may be adopted. Let us imagine a number of proteins and their modifications forming a self-sustained autonomous clock, and let us also assume that this subcellular clock is neither entrained by a major phase-locker (i.e., the cell cycle or the circadian clock) nor synchronized between different cells in culture (Figure 6B). Time-lapsed samples that are collected *en masse*, even if prepared via cell cycle synchronization or light-dark entrainment, will not be sufficient to study this cell-intrinsic clock as differential phases of rhythms in individual cells will completely obscure a meaningful average and lead to a noisy flat line (Figure 6B). To identify such a time-keeping mechanism more accurately, cells should be individually tracked instead. In support of this suggestion, a recent study has developed a microfluidics system to image and track individual bacteria for tens of consecutive generations under tight environmental conditions, successfully identifying synthetic gene oscillators – even with circuit variants that display up to a 30-fold difference in period (Luro et al., 2020). Such studies will lead the way on how we can alleviate the systems-level challenges in studying autonomous clocks.
